# Association between Sudden Sensorineural Hearing Loss and Lyme Disease

**DOI:** 10.3390/jcm10051130

**Published:** 2021-03-08

**Authors:** Klaudia Sowula, Joanna Szaleniec, Kamila Stolcman, Piotr Ceranowicz, Sebastian Kocoń, Jerzy Tomik

**Affiliations:** 1 ENT Department, Faculty of Medicine, Jagiellonian University Medical College, Jakubowskiego 2, 30-688 Krakow, Poland; sowula.k@gmail.com (K.S.); joanna.szaleniec@uj.edu.pl (J.S.); kamila.stolcman@gmail.com (K.S.); sebastian.kocon@uj.edu.pl (S.K.); 2 Department of Physiology, Faculty of Medicine, Jagiellonian University Medical College, Grzegórzecka 16, 31-531 Krakow, Poland; piotr.ceranowicz@uj.edu.pl

**Keywords:** Lyme disease, Borrelia burgdorferi, sudden sensorineural hearing loss, auditory nerve

## Abstract

Objectives: Sudden sensorineural hearing loss (SSNHL) is defined as sensorineural hearing loss of 30 dB or more over at least three adjacent audiometric frequencies occurring within a 72-h period of time. One of the causes of SSNHL could be the progressive inflammatory state caused by an infection. The aim of this study was to assess the prevalence of SSNHL caused by various factors, most importantly those potentially related to Lyme disease. Material and Methods: The study includes a group of 86 patients between the ages of 20 and 70 who were hospitalized due to SSNHL between 2017 and 2018. All of these patients underwent a detailed medical interview and an otolaryngological examination, including audiological and diagnostic tests. Additionally, ELISA and Western blot tests were performed to confirm the diagnosis of Lyme disease. Results: In this group of 86 patients, nine patients presented with positive antibodies toward Borrelia burgdorferi *sensu lato*. This group was treated with antibiotics and experienced partial or complete regression of their deafness. This may suggest a relationship between SSNHL and Lyme disease. Conclusion: Infections caused by Borrelia burgdorferi may contribute to the development of inflammatory and angiopathic lesions, which are a possible cause of SSNHL. The longer the duration of the infection, the greater the likelihood of permanent and irreversible changes in the vessels of the cochlea or auditory nerve. Therefore, serological tests for Borrelia burgdorferi should be performed during the diagnosis of SSNHL as a possible cause of this illness.

## 1. Introduction

Over 5% of the world’s population is living with a disabling hearing loss impacting on the individual’s ability to communicate with others [1]. Hearing impairment can result in significant communication disorders, leading to significant educational, social, and vocational ramifications that can adversely affect quality of life [2]. Damage to one or more parts of the hearing organs can lead to complete or partial hearing loss. This phenomenon can occur at any age and may affect one or more locations. Due to the location, the hearing loss is divided into conductive hearing loss (caused most often by inflammation and traumatic conditions of the middle ear, otosclerosis, malformations and tumors of the middle ear), sensorineural hearing loss (SNHL), and mixed hearing loss. SNHL can be a consequence of trauma, inflammation, metabolic and toxic damage, autoimmune diseases, as well as Meniere’s disease, N VIII tumors, and meningitis. A particular example of SNHL is sudden sensorineural hearing loss (SSNHL).

Sudden sensorineural hearing loss (SSNHL) is a rapidly developing (up to 72 h) hearing deterioration in one ear or less frequently in both ears. The SSNHL definition indicates that the main criterion for diagnosis is the shift of the hearing threshold with a depth of ≥30 dB in the range of at least three adjacent frequencies [3]. About 90% cases of SSNHL are idiopathic, and their cause remains unknown. That may be associated with vascular disorders, injuries, infections (particularly viral), as well as immunological or metabolic disorders.

There are three major theories of the development of sudden sensorineural hearing loss. The first of these is the vascular theory, in which blood flow disorders in the scope of the cochlea, caused by congestion or thrombus, and sometimes also by vasospasm play a predominant role. Another theory is the viral theory, which assumes that viruses can affect the state of microcirculation [4]. The last one is the autoimmune theory, which as a possible cause considers the deposition of immunological complexes leading to inflammatory changes in the vascular band [5]. Approximately 10% of SSNHL cases may be a symptom of other diseases, such as cerebellopontine tumors, stroke, or neoplasm of the central nervous system [6]. A separate group consists of patients with SSNHL caused by ongoing chronic inflammation or pathological processes affecting the hearing organ.

Patients with SSNHL mostly complain about sudden, usually unilateral hearing impairment, which is often preceded by feeling of fullness in the ear. Hearing loss is often accompanied by high frequency tinnitus. Dizziness and balance problems may also occur [7].

Due to the fact that the cause of SSNHL often remains unknown, medicine still looks for the possible etiology of this rapidly progressing process. One of the assumptions may be that infection caused by Borrelia burgdorferi is the reason of SSNHL.

Lyme disease (LD) is caused by the spirochete Borrelia burgdorferi *sensu lato* genospecies complex, which is transmitted by infected Ixodes species ticks. LD exists world-wide and is increasing in many countries, especially in Europe. In Poland, the number of reported cases of LD was 21.528 in 2017 year, and the incidence rate was 56.02/100,000 population [8].

Otolaryngological manifestation of LD included different symptoms such as sore throat, otalgia, cervical adenopathy, facial nerve palsy tinnitus, vertigo, and hearing loss. Many authors have been mentioned the occurrence of hearing loss in LD patients [3,9,10,11], but only a few reports in the literature concern sudden loss of hearing cases [9,12,13,14]. The aim of the study was to assess the prevalence of SSNHL among the hospitalized patients; furthermore, it includes an attempt to answer the question of whether the Borrelia burgdorferi infection that causes Lyme disease may be the reason for SSNHL.

## 2. Material and Methods

### 2.1. Patients

The study included eighty-six consecutive patients hospitalized due to SSNHL (49 (57%) women and 37 (43%) men) in the ENT Department of Jagiellonian University in Krakow between January 2017 and May 2018. SSNHL was defined as a sensorineural hearing loss of 30 dB or more across at least three contiguous frequencies of 500, 1000, 2000, and 4000 Hz occurring within 72 h.

The data analysis excluded patients after head injuries (due to the fact that sudden hearing loss appeared after the injury episode) and pregnant women (due to the possible impact of the hormonal economy to SSNHL). Demographic and clinical characteristics of the patients with sudden hearing loss are shown in Table 1.

All patients participated in the same detailed protocol, which included a chart review to obtain age, gender, status of the ears (normal otoscopic appearance), presence of tinnitus or/and vertigo, biochemical blood samples, cytomegalovirus (CMV), and Borrelia serological findings. Magnetic resonance imaging (MRI) was performed for patients for whom the brainstem audiometry suggested the pathology of retro cochlear space. The ELISA tests (IgM and IgG classes) were performed in order to detect infection with *Borrelia burgdorferi*. If a positive test result was obtained, the Western blot confirmation test was made in both classes. Cross-reactions may occur in the ELISA test (mainly for flagellin-p41 protein), and therefore, we confirmed it with the Western blot test. If for some reason the test result was false positive, then the patient would have to have some disease, e.g., autoimmune disease—and we excluded these during the interview. In doubtful cases, differential diagnosis with other diseases should be performed. The result should always be interpreted considering the patient’s clinical condition.

The standard treatment patients with SSNHL included corticosteroid, vasodilators, and ionotropic agents.

### 2.2. Audiological and Otoneurological Examination

The audiometric evaluation was assessed at the initial study and after 30 days by pure-tone average audiometry (PTA) on low (250, 500, 1000 Hz) and high (2000, 4000, 6000 Hz) frequencies for both ears. Moreover, each patient had an additional auditory brainstem response (ABR) and electronystagmography (ENG) examinations. The outcome data included PTA of hearing thresholds of 500, 1000, 2000, and 4000 Hz. The frequencies of bone conduction were the same as the air conduction.

The ABR test was performed on an ICS Chartr EP 200 Otometrics device using a 2–4 kHz crackle acoustic stimulus with a duration of 100 µs. A hearing threshold of ≤20 dB nHL for each ear separately was assumed as the correct result. The abnormal result was assessed for individual hearing loss thresholds: 20–40 dB nHL, 40–60 dB nHL, and ˃60 dB nHL.

The videoelectronystagmography (VENG) was performed on the Aquamatic equipment number 24510244. In the VENG study, the excitability of the labyrinths was assessed in caloric tests, assuming canal paresis (CP) ≤ 20% as the norm.

Tinnitus reported by patients was divided into high- and low-frequency tinnitus due to its frequency.

The study protocol was reviewed and approved by the Bioethical Committee of Jagiellonian University in Krakow (1072.6120.318.2018).

## 3. Results

Older patients aged 61–70 years prevailed among these patients in Poland with SSNHL for both females and males. The control group (Table 2) consisted of 36 patients (21 (58.3%) women and 15 (41.7%) men) aged 22–68 years (mean age 50.2 years) diagnosed with seropositive Borrelia burgdorferi infection using the same serological criteria as in the sudden hearing loss patient groups (Table 1). These patients did not have SSNHL or other symptoms indicative of damage to the organ of hearing and/or balance. IgM antibodies were found in 17 (47.2%) patients, IgG antibodies were found in 14 (38.8%) patients, and both IgM and IgG were found in the remaining 5 (14%) patients.

**Figure 1:**
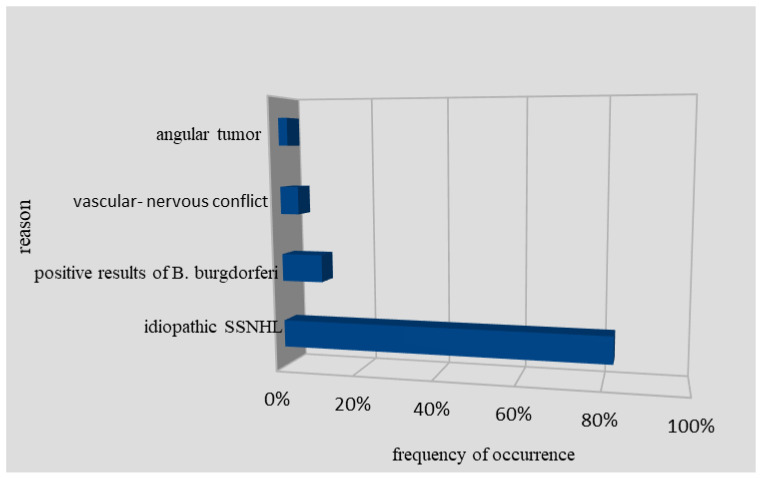
Reasons for sudden deafness in the examined group of patients (AT—two patients, VNC—four patients, PRBB—nine patients, ISSNHL—71 patients).

Among 86 SSNHL patients, there were also two people with detected cerebellopontine tumors and four people with neurovascular conflicts as a potential cause of SSNHL (Figure 1). After thorough diagnosis and deep analysis of available studies, it was found that in 71 patients (82.5%), it was impossible to determine the exact etiology of SSNHL. However, these patients had a therapeutic response in terms of hearing improvement to parenteral corticosteroid treatment [15]. The analysis of PTA on low and high frequency in 71 patients shows respectively 68 ± 22.4 dB HL and 81.2 ± 25.7 dB HL. After the applied treatment, PTA shows respectively 48.8 ± 24.1 dB HL and 66.4 ± 32.0 dB HL. Six patients with SSNHL had no hearing improvement in response to corticosteroids.

In nine (10.5%) patients, the presence of positive antibodies toward Borrelia burgdorferi was detected. The PTA analysis of low and high frequency in this group of patients shows respectively 82 ± 25.4 dB HL and 88.2 ± 28.7 dB HL. After the treatment, PTA shows respectively 58.8 ± 20.1 dB HL and 60.4 ± 18.0 dB HL.

In two patients, the ABR test showed a hearing impairment reaching 20–40 dB nHL, in six patients, it was 40–60 dB nHL, and in one patient, it was over 60 dB nHL. The follow-up test revealed the ABR test result to be within the norm, and in four patients, hearing impairment reaching 20–40 dB nHL was recorded.

In seropositive patients, abnormalities in the ENG test such as reduced reactivity of labyrinth was detected in three patients (33.3%), while in one patient, canal paresis (CP) was 20–40%, in two patients, CP = 40–60%. In the control study, the VENG conducted 6 months after the treatment showed no labyrinth pathology in two patients, but one patient had CP = 20–40%.

In seropositive patients, high-frequency tinnitus occurred in four patients (two men and two women), and in one woman, tinnitus was of low frequency.

The group of patients with positive antibodies toward LD consisted of 66.7% women and 33.3% men. In this group, in 55.6% of patients (five people), there were found positive IgM antibodies with the dominant outer surface protein (OspC). In 22.2% (two patients), positive IgG antibodies with dominant p100 and VlsE antigens were found. In another 22.2% (two patients), positive antibodies for both IgM and IgG were observed. Patients with positive antibodies toward LD did not respond to intravenous corticosteroids, microcirculatory drugs, or ionotropic drugs, which activate ion channels (Lidocaine). Due to a lack of improvement after the applied course of treatment, patients were discharged from the Otolaryngology Department, referred to infectious diseases specialist, and sent to the outpatient clinic for recommended control examination after one month. Seven patients reported to a control visit. A standard dose of doxycycline was applied to four of them in a three-week period of time (2 × 100 mg per day) (Table 3).

According to the patients’ interviews, this medication contributed to the improvement of hearing. However, it did not result in a complete recovery. Tonal audiometry in four patients has shown that the hearing threshold was raised by an average of 10 dB compared to the curve recorded during hospitalization. In the remaining three patients, infectious disease specialists ordered parenteral administration of ceftriaxone (dose of 2 g per day) for a maximum period of three weeks (one patient was treated for three weeks, the other two was treated for two weeks). Those three patients reported a complete recovery of hearing (PTA shows respectively 15.20 dB HL for low frequency and 28.35 dB HL for high frequency). However, two of them complained of high-frequency tinnitus accompanying sensory-nerve hearing loss. In these patients, tinnitus was present from the beginning of the disease. In the performed tonal audiometry, a typical age curve was found.

## 4. Discussion

In this study, we wanted to determine the prevalence and association between Lyme disease and SSNHL. Based on our results, we conclude that sudden sensorineural hearing loss is an idiopathic process in approximately 90% of cases. The results of other similar articles showed the prevalence of Lyme disease to be between 0 and 21% [9,10,12,16]. In our study, it was 10.5% of patients. The true percentage may vary depending on the country, concentration of ticks, and climate. As the detection of Lyme disease increases in many countries, the possibility of performing a test for Borrelia burgdorferi infection should be considered in patients with SSNHL. This test may be helpful in the diagnosis of this disease, especially when there are no other obvious causes of hearing loss and/or vestibular symptoms.

Borrelia burgdorferi infection may contribute to the development of inflammatory and angiopathic lesions, which are a possible cause of SSNHL. The longer the duration of the infection, the greater the likelihood of permanent and irreversible changes in the cochlea vessels or auditory nerve. Positive serological tests toward Borrelia burgdorferi infection with immediate proper treatment may also prevent the development of other serious complications of long-lasting Lyme disease [11,17].

However, nowadays, scientists’ opinions about the contribution of Borrelia burgdorferi to this complication are not unanimous due to receiving controversial data as a result of independent investigations, which were conducted in different countries [18]. In the USA, Borrelia burgdorferi is the most common among bacterial causes of sensorineural deafness [19]. In the research conducted by Lorenzi et al., among 47 consecutive patients with sudden deafness, antibodies in serum against Borrelia burgdorferi were detected in 10 (21.3%) by an enzyme immunoassay test (EIA), and this was confirmed by Western blot test [9]. In the research study conducted by M. Peltomaa, IgG antibodies against Borrelia burgdorferi were found in 20 (12.1%) of 165 patients with SSNHL. In this study, a positive result in PCR was revealed in two, and migrating erythema was observed in two individuals [10].

In the serological diagnosis of Lyme disease, various limitations should always be considered. Lyme disease diagnosis does not solely rely on the result of a serological test or of a PCR test. Clinical and epidemiological characteristics should be taken into consideration in the test interpretation. The predictive value of the serological test depends on the sensitivity and specificity of the test and on the prevalence; a positive serology in a patient who do not present with any objective lesions suggestive of Lyme disease has a predictive value close to zero [20]. The most frequent limitations and pitfalls of serology are cross-reactions, false IgM positivity, a seronegative window period at the early time of the infection, and serologic scars with a suspicion of reinfection [21].

There are also studies that report on an indicated low incidence of seropositive results in patients with sensorineural deafness [22,23]. In the Swiss [16] retrospective investigation, which included patients with sensorineural deafness, antibodies against Borrelia burgdorferi were detected only in one patient. However, after further examination of the patient, the diagnosis of LD was excluded. Similar results were obtained by group of researchers led by Hydén et al. In this study, antibodies against Borrelia burgdorferi were detected in blood serum only in four out of 21 patients. In an investigation of cerebrospinal fluid, antibodies against Borrelia burgdorferi were not detected in any patients, hence the conclusion that diagnosis of LD in these patients was doubtful [12].

Single cases of sensorineural hearing loss in patients with LD are found in the scientific literature. A case of bilateral sensorineural deafness and spastic paraparesis in association with LD has been recorded. In this report, despite the absence of data concerning tick bite, the contribution of Borrelia burgdorferi to the development of the complication was confirmed by positive results of blood serum and blood alcohol tests via EIA, dot-blot, and ELISA [22].

In another report, a case of coincidence of LD and sensorineural deafness development has been described. In this case, the diagnosis of LD was confirmed by the detection of IgM against Borrelia burgdorferi in blood serum by an EIA test and Western blotting. IgG antibodies against Borrelia burgdorferi were not detected. The therapy involved the use of an antibiotic (doxycycline) with corticosteroids and hyperbaric oxygenation [13,24,25,26]. In another study, a case of LD was complicated by rapid sensorineural deafness and paralysis of the facial nerve. Antibodies against Borrelia burgdorferi were detected in the patient’s blood serum by the EIA test and Western blotting. In this case, the patient received therapy with ceftriaxone. Signs of nerve facialis lesion were regressing, but hearing was not recovered [26,27]. On the other hand, Selmani et al. suggest that hearing loss in LD could be a result of inflammation impact by an immunological reaction in the cochlea by BB infection. The first manifestation of this reaction is an elevated level of IgM [14].

An extremely important observation is fact that sensorineural deafness was not preceded by migrating erythema in all cases presented in the literature, which is a pathognomonic symptom of LD and definitely confirms the etiological role of Borrelia burgdorferi in lesions of the organ of hearing. It should also be clearly stated that in most investigations, the detected diagnostic level of antibodies against Borrelia burgdorferi in patients with sensorineural deafness was not compared with the population’s seropositivity level in a particular region [18].

In conclusion, SSNHL can be associated with LD; however, it is a rare symptom. We recommend serologic testing for LD in patient with SSNHL, especially in cases with a history of tick bites, erythema migrans, and neurological, and otolaryngological symptoms. The patients with seropositive tests should be treated to prevent further complications of disease. These results have to be confirmed in more patients.

## Figures and Tables

**Table 1:** Demographic and clinical characteristics of the patients with sudden hearing loss.

	Seropositive Patients (*n* = 9)		Seronegative Patients (*n* = 77)
	Male with LD (*n* = 3)	Female with LD (*n* = 6)	
Male/female	3/0	0/6	34/43
Mean Age (Years, Range)	47.3 (31–59)	48.3 (29–62)	50.4 (22–67)
Age range (%)			
20–30	0	1 (16.6)	8 (23.5)/9 (21)
31–40	1 (33.3)	0	6 (17.6)/6 (14)
41–50	0	2 (33.3)	2 (5.9)/4 (9)
51–60	2 (66.6)	2 (33.3)	7 (20.6)/10 (23)
61–70	0	1 (16.6)	11 (32.4)/14 (33)
Side of Hearing Loss			
Right (%)	2 (66.6)	3 (50)	18 (53)/20 (47)
Left (%)	1 (33.3)	3 (50)	14 (41)/22 (51)
Bilateral (%)	0	0	2 (6)/1 (2)
Tinnitus (%)	2 (66.6)	3 (50)	26 (76.5)/34 (79.1)
Vertigo (%)	1 (33.3)	2 (33.3)	8 (23.5)/12 (27.9)

**Table 2:** Demographic and clinical characteristics of the control group patients.

	Seropositive Patients (*n* = 36)	
	Male with LD (*n* = 15)	Female with LD (*n* = 21)
Male/Female	15/0	0/21
Mean Age (Years, Range)	45.8 (23–60)	45.8 (23–60)
Age range (%)		
20–30	1 (6.66)	2 (9.52)
31–40	3 (19.98)	5 (23.8)
41–50	6 (39.96)	8 (38.08)
51–60	3 (19.98)	6 (28.56)
61–70	2(13.32)	
Serology		
IgM(+), IgG(−) (%)	8 (22.2)	9 (36.0)
IgM(−), IgG(+) (%)	6 (16.6)	8 (22.2)
IgM(+), IgG(+) (%)	2 (5.5)	3 (8.3)

**Table 3:** Summary of laboratory and clinical results in patients with Lyme disease (LD) and SSNHL.

Patient Serology	ABR(dB) Tinnitus	VENG (CP%)	Response to Treatment
1. IgM(+), IgG(−)	20–40 None	Normal	Dox. H imp.
2. IgM(+), IgG(−)	˃60 Left	40–60	Dox. H persist.
3. IgM(+), IgG(−)	40–60 None	Normal	Dox. H persist.
4. IgM(+), IgG(−)	40–60 None	40–60	N/A
5. IgM(+), IgG(−	20–40 Right	Normal	Ceftriax. H imp.
6. IgM(−), IgG(+)	40–60 Bilat	Normal	Ceftriax. H imp.
7. IgM(−), IgG(+)	40–60 Right	Normal	N/A
8. IgM(+), IgG(+)	40–60 Bilat	20–40	Ceftriax. H.imp.
9. IgM(+), IgG(+)	40–60 None	Normal	Dox. H persist.

Dox.—Doxycycline, Ceftriax.—Ceftriaxone, H—hearing, imp.—improved, persist.—persistent, N/A—no data.

## Data Availability

All relevant raw data from the data presented in the manuscript are available from the authors of the study upon request.
